# Eccentric contractions during downhill running induce Osgood‒Schlatter disease in the tibial tuberosity in rats: a focus on histological structures

**DOI:** 10.1038/s41598-023-36914-7

**Published:** 2023-06-18

**Authors:** Hirai Suito, Kaoru Fujikawa, Masafumi Ohsako

**Affiliations:** 1 Graduate School of Human Life Design, Toyo University, 1-7-11 Akabanedai, Kita-Ku 115-8650, Tokyo, Japan; 2 Japan Society for the Promotion of Science Research Fellowships DC, Tokyo, Japan; 3 Department of Oral Anatomy and Developmental Biology, Showa University School of Density, Tokyo, Japan; 4 Graduate School of Health and Sports Science, Toyo University, Tokyo, Japan

**Keywords:** Musculoskeletal system, Anatomy, Pathogenesis

## Abstract

Osgood–Schlatter disease (OSD), a condition that affects adolescents, causes inflammation, pain, and prominence at the tibial tuberosity. The causes of OSD are not well understood, but eccentric contractions in the quadriceps have been suggested as a possible factor. To investigate this, a study was conducted in which 24 rats were divided into two groups: the downhill treadmill running (DR) group and the control (CO) group. The DR group underwent a preliminary running program for 1 week, followed by a main running program for 3 weeks. The results showed that the deep region of the tibial tuberosity in the DR group was larger than that in the CO group, and inflammatory cytokines involved in gene expression were upregulated in the DR group. The anterior articular cartilage and deep region in the DR group were also immunoreactive to substance P. Additionally, high-activity chondrocytes of small size were observed in the non-calcified matrix. Thus, the DR group exhibited symptoms similar to OSD, including inflammation, pain, and prominence. These findings suggest that eccentric contractions in the quadriceps may play a role in the development of OSD. Further research is needed to better understand the pathophysiology of this condition and develop effective treatment options.

## Introduction

Osgood‒Schlatter disease (OSD) commonly occurs at the knee joint^1^, and its symptoms include prominence, pain, and inflammation in the tibial tuberosity^2^. Previous studies reported that this pathological condition can persist for a long time in adolescent boys^3^ and can significantly influence exercise tolerance and activities of daily living^4^. The quadriceps is embedded in the tibial tuberosity, and it extends the knee joint by contracting. OSD is induced in the tibial tuberosity through repetitive eccentric contractions of the quadriceps causing overactivity of the knee joint, and it has been revealed that many kicking or landing movements can also induce OSD^5,6^. Therefore, OSD is particularly common in active adolescent boys. Reduced flexibility of the quadriceps and triceps surae can also cause OSD, which leads to increased strain and strong contraction at the tibial tuberosity.^2^. In addition, the point at which a tendon or ligament attaches to the bone is known as an enthesis, and the tibial tuberosity is one such example^2,7^, which can be observed in the fibrocartilage. In general, sports injury to an enthesis is called enthesopathy^8^. Inflammation of the supraspinatus insertion site is another example of an enthesopathy that occurs at the greater tubercle and can lead to an increase in the fibrocartilage portion^9^. Histological findings of the tibial tuberosity in OSD have mostly been reported based on macroscopy^10,11^. Therefore, the histological features based on microscopic analyses are not adequately understood^7^.

A previous study observed the histology of rat tibial tuberosity and categorized the superficial and deep regions by the different histological structures^8^. In brief, the superficial region is embedded in the patellar tendon fibers, and with aging, there is high calcification of the patellar tendon fibers which become embedded into the superficial region. The enthesis is divided into four zones: tendon, non-calcified fibrocartilage, calcified fibrocartilage, and bone^12^. According to a previous study, overuse activities with eccentric contractions increase the portion of fibrocartilage in the enthesis^9^. In the quadriceps, eccentric contractions occur during downhill running (DR), leading to a stronger pull on tibial tuberosity compared with that during concentric contractions^13^, therefore influencing the histological structures on the enthesis. In contrast, the deep region of the tibial tuberosity comprises non-calcified hyaline cartilage. Immature chondrocytes are observed in deep regions during the early growing period, while hypertrophic chondrocytes are observed during the remainder of the growing period^14^. With age, hyaline cartilage is not observed in the deep region, but bone tissue forms in the same region. The growth plate is structured similarly to the deep region and is called the resting and proliferation layer. Chondrocytes in the growth plate undergo skeletal growth through cell multiplication in the proliferation layer^15,16^. Thus, the importance of examining chondrocytes in the deep layer for understanding the characteristics of the tibial tuberosity is crucial. Cartilage tissue frequently develops during inflammation due to excessive mechanical stress, along with inflammatory factors participating in interleukin-6 (IL-6) expression^9^. According to previous research, prostaglandin E2 (PGE2) is induced by the expression of inflammation cytokines^16,17^. Initially, cyclooxygenase 2 (Cox2) converts arachidonic acid to prostaglandin H2 (PGH2) during inflammation, and PGH2 is synthesized to PGE2 by Ptges^16^. In general, the maturation of chondrocytes stems from the hypertrophy of immature chondrocytes^18–20^. However, PGE2 has been shown to suppress chondrocyte differentiation^21^ as well as calcification in the cartilage matrix. Based on these facts, OSD induces inflammatory cytokines and PGE2 synthesis, and it is speculated that this influences the differentiation and calcification in the tibial tuberosity. However, this hypothesis remains unsubstantiated due to the histological structure and pathological future of OSD remaining unclear. This problem can be resolved by creating an animal model for OSD; however, such a model has not yet been developed.

The present study aimed to recreate the structure of OSD from the viewpoint of eccentric contraction of the quadriceps in rats and the pathogenesis of OSD. We hypothesized that OSD could be reproduced by DR. In addition, we hypothesized that the prominence of the tibial tuberosity in OSD results from the proliferation of chondrocytes in the deep regions.

## Methods

### ARRIVE guidelines

This study was conducted in accordance with Essential 10 within ARRIVE guidelines 2.0. Details of each item are given below. Moreover, we confirmed that all experiments were performed in accordance with relevant guidelines and regulations.

### Study design, sample size, and outcome measures

This study aimed to reproduce the histological structure of OSD using downhill running exercise and was analyzed in two groups: a downhill running group and a control group. Moreover, the animal species, number of animals in each group, and number of animals kept in one cage are listed in the “Experimental design” section. A goal of this study was to ensure the dignity of the animals and the reliability of the research results. The experimental protocol was approved by the Committee of Animal Experiments and Ethics for Research at the Graduate School of Life Design, Toyo University, Tokyo, Japan (approval no.: 2019-04).

### Inclusion and exclusion criteria, and randomization

All rats used in this experiment were included in the analysis. The right leg of all rats was used for histological analysis (n = 12), while the left leg was used for bone morphometry and gene expression analysis (n = 6). Additionally, the groups in this study were assigned using a randomization technique in which cards with an irregular arrangement of numbers from 1 to 24 were drawn, and 12 cards were assigned to each group. The rats were then sorted according to the numbers on the cards.

### Blinding

Hirai Suito conducted the experiment and performed the initial evaluation of the results, the results were then shared with Kaoru Fujikawa and Masafumi Ohsako while keeping the group names hidden.

### Statistical measures

Statistical analysis in this study was performed IBM SPSS Statistics ver.26. In addition, we employed a t-test with no correspondence in the test between the two groups. Therefore, all *n* numbers for both groups were unified during testing.

### Experimental procedures

We utilized male Wistar strain rats, and 6-week-old rats were used in this study due to their correspondence to the adolescent stage in humans. The rats were brought in from the Nippon Bio-Supply Center in a pathogen-free state but were not genetically modified for exercise experiments. Body weights were measured at the end of the experiment and are listed in Table 1 along with SD (Table 1).

**Table 1:** Rat status in this study.

Group name	Microbiological status	Body weight ± SD (g)
Control group	Specific-pathogen-free	283.6 ± 7.43
Downhill running group	Specific-pathogen-free	297.6 ± 8.55

### Experimental animals

**Figure 1:**
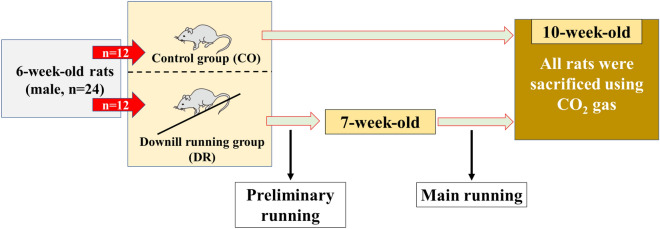
The simplified schematic diagram in this study.

We conducted a running exercise experiment using a treadmill (MK-690) manufactured by Muromachi Kikai Co. The detailed protocol and the reports used as references are described in the "Experimental design" section. A simplified schematic diagram of the experimental procedure is shown in Fig. 1. In addition, the experiment was conducted at the Asaka Campus of Toyo University, which is approximately 7 m above sea level. Furthermore, all statistical analyses included a p-value.

### Experimental design

A total of 24 rats (6-week-old, Wistar strain, male) were divided into the downhill treadmill running (DR) group (n = 12) and the control (CO) group (n = 12) (Nippon Bio-Supply Center, Tokyo, Japan), randomly. Up to four rats were housed per cage and provided water and food ad libitum (Oriental Yeast Co., Ltd., Tokyo, Japan). The running protocol used in this study is based on the experimental protocol of Soslowsky et al.^22^. The purpose of this study was to develop an overuse rat model of OSD. The DR protocol consisted of preliminary running for 1 week and a main running program for 3 weeks. The preliminary running conditions were as follows: speed, 10 m/min; angle, − 10°; time, 30 min once; and frequency, 5 days/week. The main running program conditions were as follows: speed, 17 m/min; angle, − 15°; time, 60 min once; and frequency, 5 days/week. At the end of the experimental period, all rats were euthanized using CO_2_ gas, the proximal tibia was fixed using 4% paraformaldehyde, and sagittal sections were observed on various specimens.

### Non-decalcification specimens

The non-decalcified specimens were embedded in Rigolac resin (Nisshin EM, Tokyo, Japan) for the observation of calcification conditions and were stained with toluidine blue^23^. Morphometry was performed on 2-hydroxyethyl methacrylate (GMA) resin specimens, which were sectioned at 4 µm on the sagittal plane.

### Paraffin-embedded sections and general stain

The specimens were decalcified using an ethylenediamine tetra acetic acid solution for 3 weeks. Thereafter, the specimens were embedded in paraffin. After staining with safranin O^24^, paraffin-embedded sections were observed using light microscopy.

### Immunohistochemistry staining

First, the paraffin was removed by immersion in xylene for 1 h in a wet box. Next, endogenous peroxidase was removed using 0.3% H_2_O_2_ in methanol for 15 min, and hyaluronan on the specimen was treated with hyaluronidase (37 °C, 2 h). Furthermore, sections were blocked with 3% bovine serum albumin for 30 min. Substance P (Abcam, Cambridge, UK) (× 300) was treated with 0.3% proteinase K for 15 min for immunoreactions, and the primary antibody was incubated on specimens overnight at 4 °C. The specimens were observed for immunoreactivity using a light microscopy.

### Morphometry in the tibial tuberosity

Morphometry of the tibial tuberosity was measured in the sagittal plane section in the GMA specimens after toluidine blue staining^25^. In this study, the whole, superficial and deep regions, and the thickness of the periosteum were measured. Moreover, the superficial region was histologically classified into three portions: patellar tendon, fibrocartilage, and calcification. All specimens were measured from the center of the tibia, and measurement was performed after histological confirmation that the anterior cruciate ligament was macroscopically visible and that there were no differences in the structure of the chondrocyte column of the epiphyseal plate. Morphometry measurements in this study were taken for the “area,” “thickness,” and “cell number” in the tibial tuberosity using WinROOF Version7.4.0 (Mitani Corporation, Hukui, Japan).

### Quantitative real-time polymerase chain reaction

First, the tibial tuberosity was extracted for total RNA extraction using TRIzol (Thermo Fisher Scientific, Tokyo, Japan). To avoid mixing with the total RNA of other tissues, a notch was made in the periosteum below the tibial tuberosity, and only the tibial tuberosity was removed. cDNA was synthesized using the iScript gDNA Clear Synthesis Kit (Bio-Rad, Hercules, CA, USA). Real-time PCR was performed using a CFX96 Real-Time System (Bio-Rad) with a TaqMan probe (Thermo Fisher Scientific). The PCR conditions were as follows: initial denaturation at 95 °C for 20 s, followed by 40 cycles of denaturation at 95 °C for 15 s, and a final extension at 60 °C for 1 min. GAPDH mRNA levels were quantified as an internal control. The relative expression levels were calculated using the Δ (ΔCT) method^26^.

### Statistical analyses

All data are expressed as mean ± standard deviation. Statistical analysis was performed using the t-test. A p-value of < 0.05 was considered statistically significant. Furthermore, all statistical analyses were performed using IBM SPSS Statistics ver.26.

## Results

### Histological characteristics of each region of the tibial tuberosity

**Figure 2:**
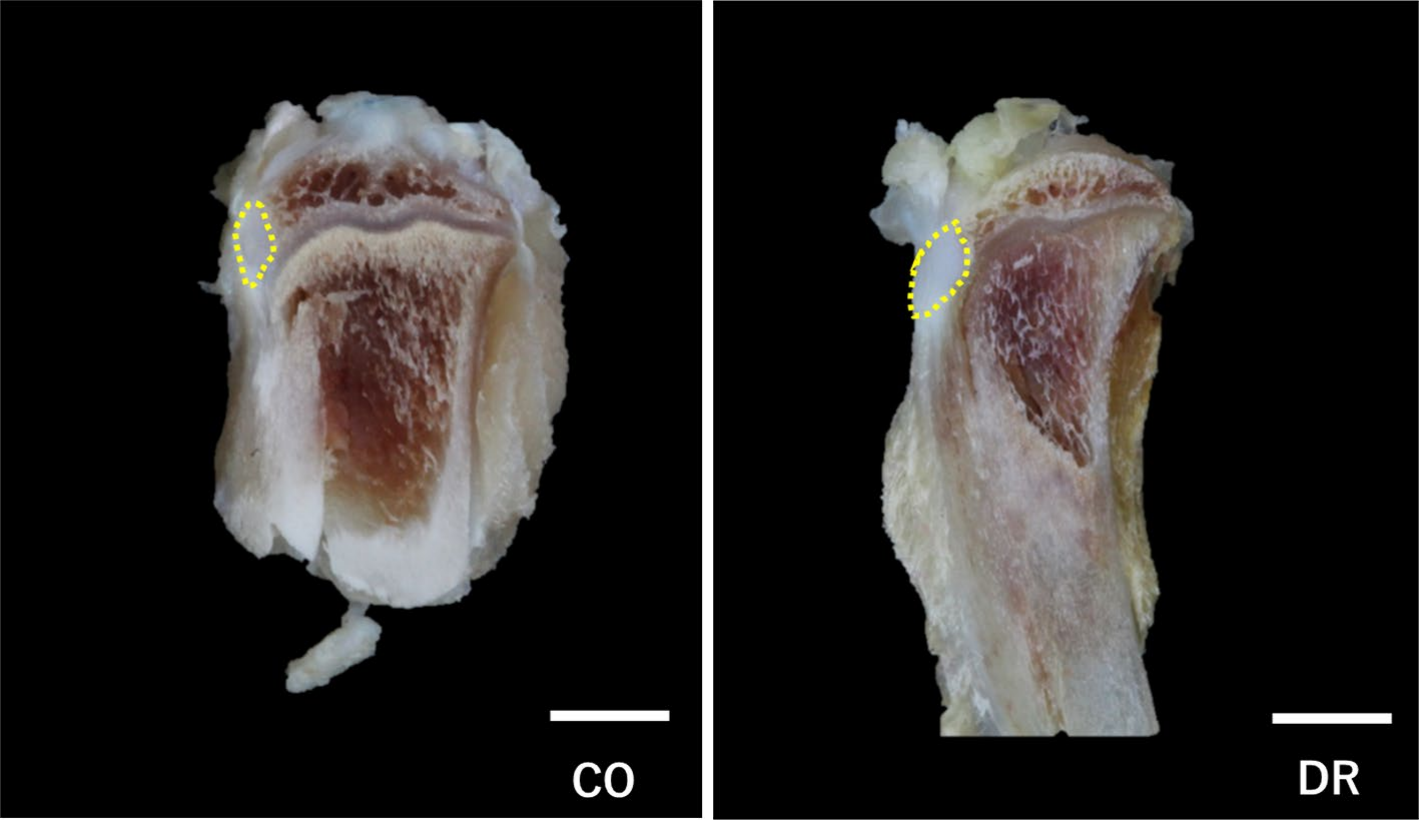
Macroscopic observation of the tibial tuberosity (bar = 3 mm, macro specimen). The tibial tuberosity (yellow cycle) is observed in the frontal epiphyseal tibia. CO group, control; DR, downhill running.

The tibial tuberosity was observed in the frontal epiphyseal tibia, and the tibial tuberosity in DR was larger CO (Fig. 2).

**Figure 3:**
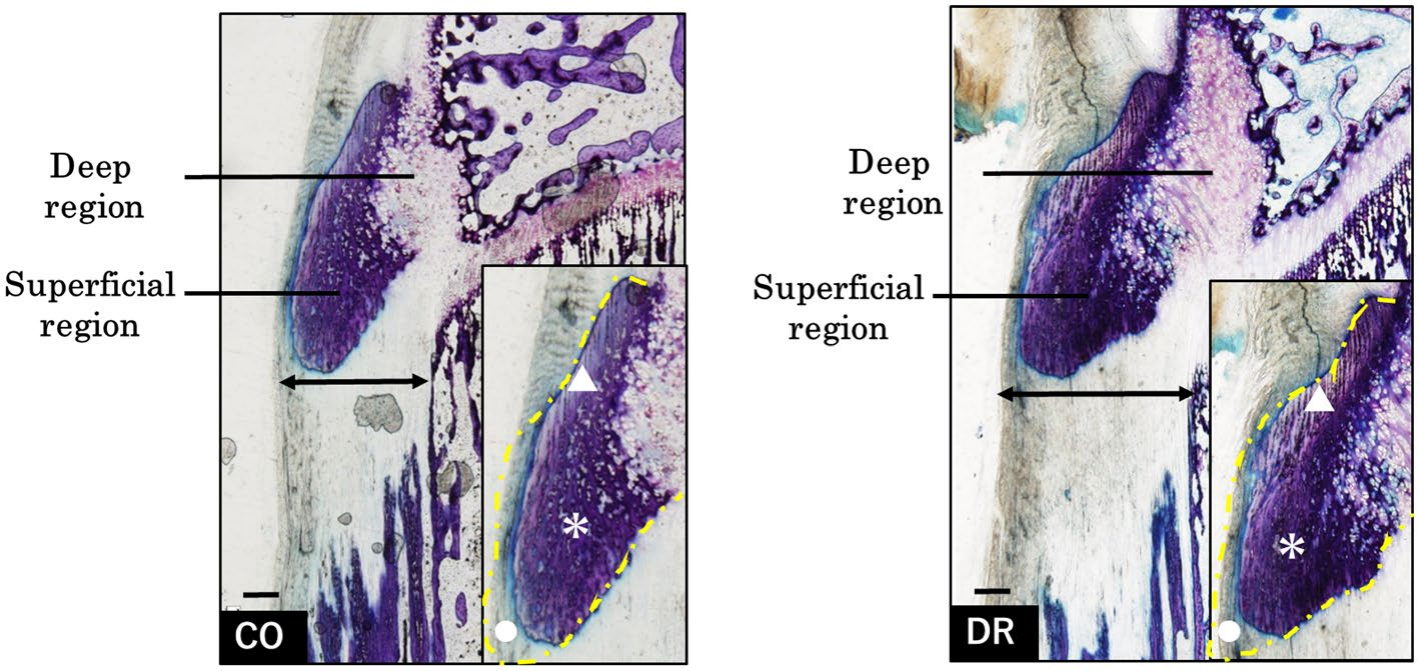
Basic structures in the tibial tuberosity (bar = 100 µm, non-decalcification specimen in toluidine blue). The tibial tuberosity comprises the deep region (structuring non-calcified cartilage tissue) and the superficial region (embedding the patellar tendon). Magnification of the superficial region, fibrocartilage (▲), calcification (*), and patellar tendon (●) portions. The periosteum (arrow) is located under the tibial tuberosity, and its thickness is greater in DR than in CO. CO group, control; DR, downhill running.

The tibial tuberosity of both groups was divided into two regions, deep and superficial, based on toluidine blue staining. The deep region comprised chondrocytes and a metachromatically stained cartilage matrix. In contrast, the superficial region was histologically classified based on its staining with toluidine blue. The patellar tendon portion comprised only the patellar tendon, which was not stained with toluidine blue. The fibrocartilage portion was the insertion site of the patellar tendon, which was lightly stained with toluidine blue. The calcification portion was highly calcified, and was not stained with toluidine blue (Fig. 3).

**Figure 4:**
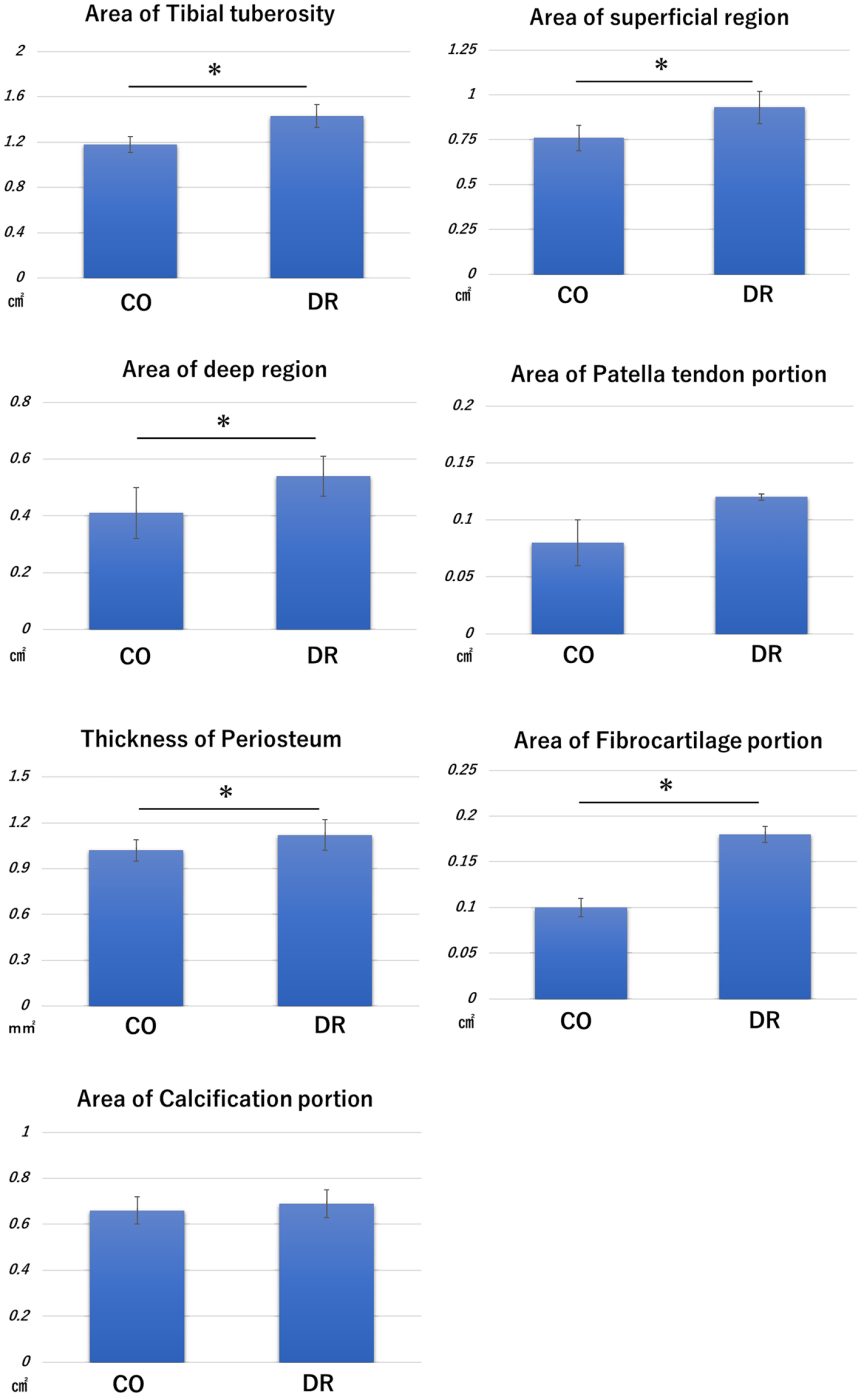
Results of morphometry. *Indicates a p-value of < 0.05. CO group, control; DR, downhill running.

The fibrocartilage portion in the control group (CO) was observed to be smooth and thin, while that of the DR was rough and thick, with the anterior portion of the fibrocartilage portion being the most prominent. Morphometry revealed significant differences between the CO and DR groups in terms of the fibrocartilage portion area, as well as other morphometric measurements (Fig. 4).

**Figure 5:**
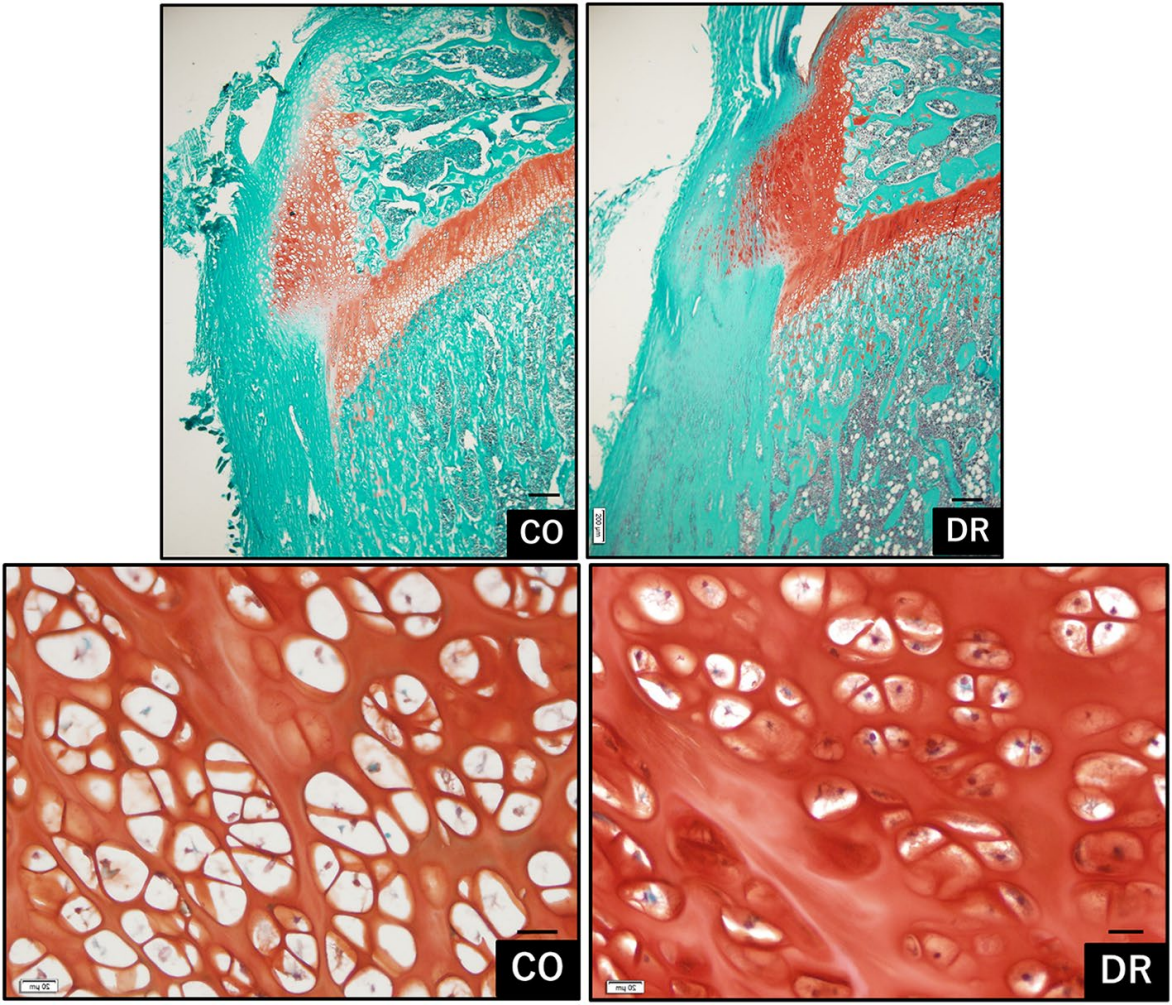
Comparison of cell structures in the deep region (bar = 200 µm for upper row and 20 µm bottom row, decalcification for paraffin sections in safranin O). Safranin O staining was not observed in most sections of both groups. Several hypertrophic chondrocytes are present in the deep region of the CO, while in contrast, small chondrocytes are observed in the deep region of DR. CO group, control; DR, downhill running.

Moreover, the prominence of the tibial tuberosity was higher in the DR group than that in the CO group, and the periosteum under the tibial tuberosity was thicker. Safranin O staining for cartilage observation revealed that the deep region was dyed in each group, but the superficial region was not. The anterior deep region for safranin O staining showed enlargement with hypertrophic chondrocytes mostly observed in CO, however DR mostly had small-type chondrocytes (Fig. 5).

**Figure 6:**
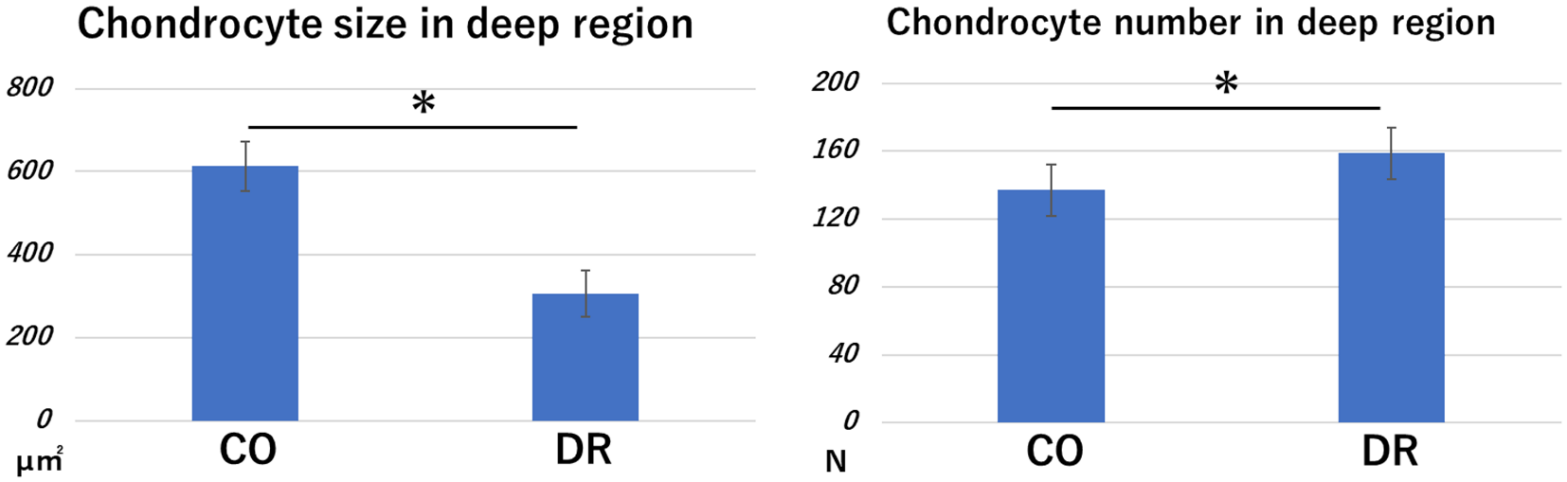
Morphometry of chondrocytes in the deep region. The chondrocyte size in CO was significantly greater than that in the DR, but the chondrocyte number in CO was significantly lower than that in the DR. CO group, control; DR, downhill running.

The size of chondrocytes in the deep region in CO was significantly greater than that in the DR (p = 0.000009), but the chondrocyte number in the deep region in the CO was significantly lower than that in DR (p = 0.05) (Fig. 6).

### Morphometry results of the tibial tuberosity

The areas of the whole (p = 0.005), superficial (p = 0.037), and deep region**s** (p = 0.04) in the tibial tuberosity of the DR were significantly higher than those of the CO (Fig. 4). In addition, the fibrocartilage portion was significantly different between the DR and CO (p≦0.0001), but the calcification portion (p = 0.577) and patellar tendon portion (p = 0.057) were not. Furthermore, DR had a significantly thicker periosteum than CO (p = 0.013).

### Immunolocalization of pain and gene expression analysis

**Figure 7:**
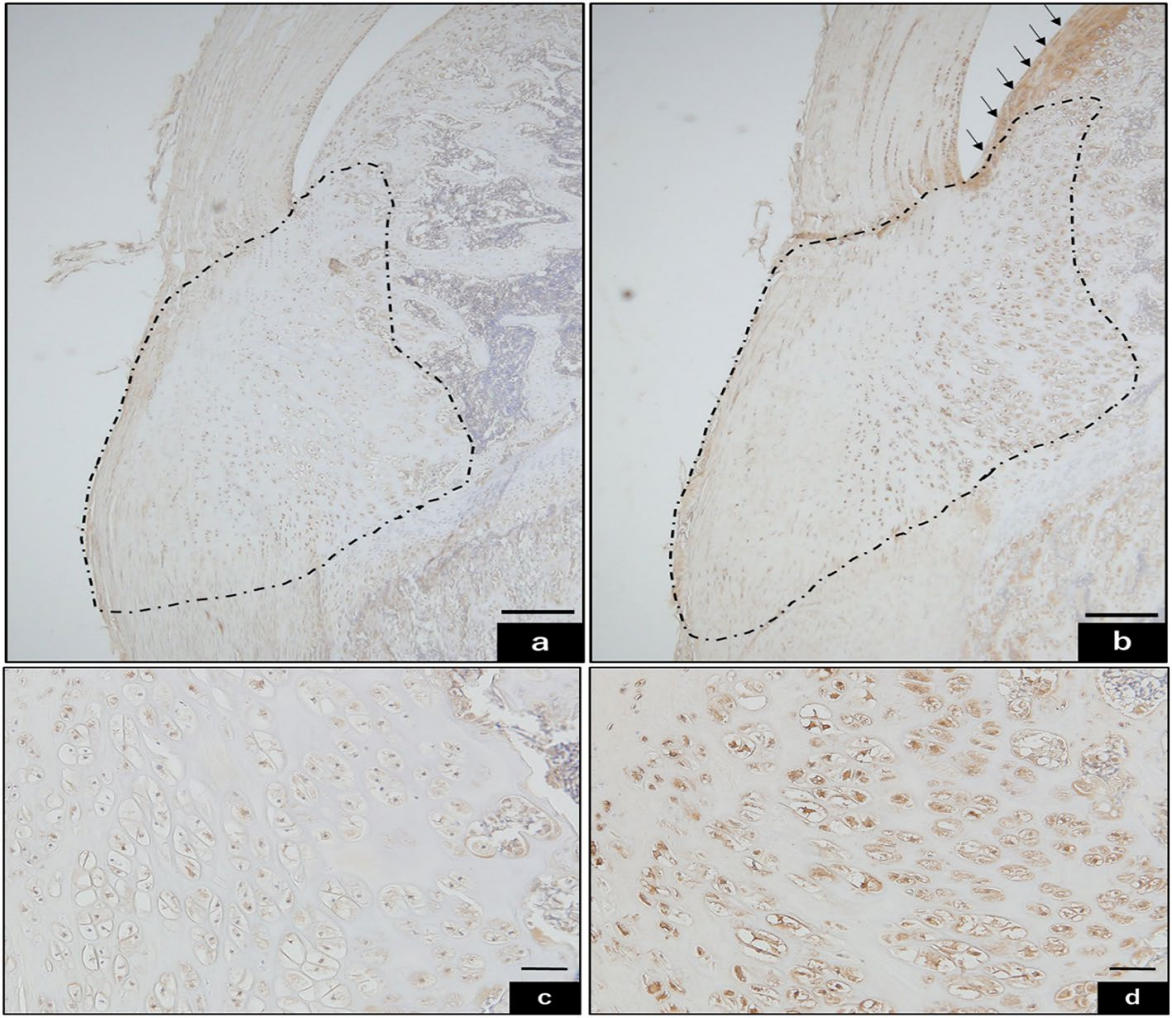
Immunohistochemistry was used to localize substance P. Bar sizes were 200 µm for (**a**) and (**b**), and 50 µm for (**c**) and (**d**). Decalcification was performed for paraffin-embedded sections. Substance P immunoreaction in CO (**a**, **c**) is observed in the deep region, but DR (**b**, **d**) is more strongly stained than CO. Additionally, staining for DR revealed the presence of anterior articular cartilage (indicated by arrows). CO group, control; DR, downhill running.

Substance P was used to identify nociceptive fibers. The articular cartilage above the tibial tuberosity was strongly immunoreactive to substance P in the DR group. In contrast, the same part was weakly immunoreactive in the CO group (Fig. 7a, b). Immunoreactivity to substance P was observed in the deep region in both groups, but the immunoreaction in the DR group was more remarkable (Fig. 7c,d).

**Figure 8:**
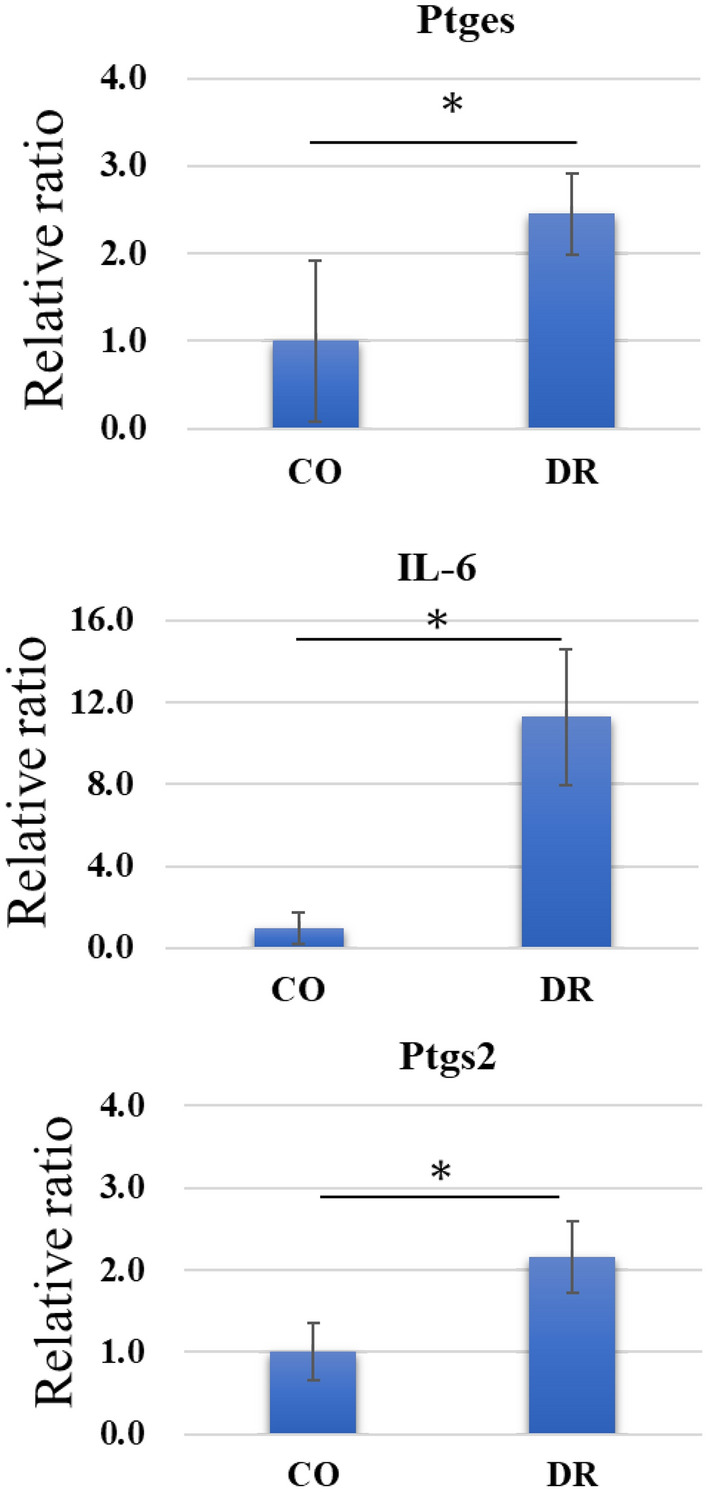
Gene expression. Real-time PCR for Ptges, Ptgs2, and IL-6 mRNA expression levels in DR are significantly higher than those in CO. CO group, control; DR, downhill running: IL-6, interleukin-6; PCR, polymerase chain reaction.

Gene expressions of Ptges (p = 0.033) and Ptgs2 (p = 0.028, involving PGE2 synthesis) were significantly more upregulated in the DR group than in the CO group. In addition, the gene expression of inflammatory cytokines was conspicuous in the DR group (p≦0.001, Fig. 8).

## Discussion

Insertion sites to bone or cartilage tissues from the muscle, tendon, and ligament are known as entheses. It is well established that the enthesis type differs between bone and cartilage insertion sites and is categorized into "fibrous" or "fibrocartilaginous" types^12^. The fibrocartilaginous type is embedded into the bone through the fibrocartilage^27^. These reports suggest that histological observation of the insertion sites is essential for understanding the structural characteristics of the enthesis, and for the fibrocartilaginous type specifically, this can help our understanding of the fibrocartilage portion at the insertion site of the tibial tuberosity. A previous study regarding the enthesis revealed that the fibrocartilage numbers increased by overuse and misuse activities (eccentric contraction)^9^. The superficial and deep regions of the tibial tuberosity were significantly higher in the DR group than those in the CO group. Notably, the fibrocartilage portion in the superficial region was the only part that underwent structural changes; thus, the impact of quadriceps contraction on the tibial tuberosity is manifested in the fibrocartilage portion. The tibial tuberosity was confirmed as an indirect type, and contractions during DR were speculated to influence the histological structures throughout the tibial tuberosity.

The beginning of the contraction force of the quadriceps is transmitted to the fibrocartilage portion, and the force transmitted to it increases with more weight-bearing during development^23^. The enthesis not only functions to disperse contraction force but also acts as a transitional zone between tendons or muscle fibers and bone, preventing direct attachment^23,28^. The histological structures of the fibrocartilage in the CO group were thin and smooth; in contrast, those in the DR group were thick, with the anterior portion being the most prominent. Based on these results, it is assumed that the anterior fibrocartilage portion is able to resist the strong contraction of the patellar tendon and the mechanical stress caused by the DR load and quadriceps contractions.

**Figure 9:**
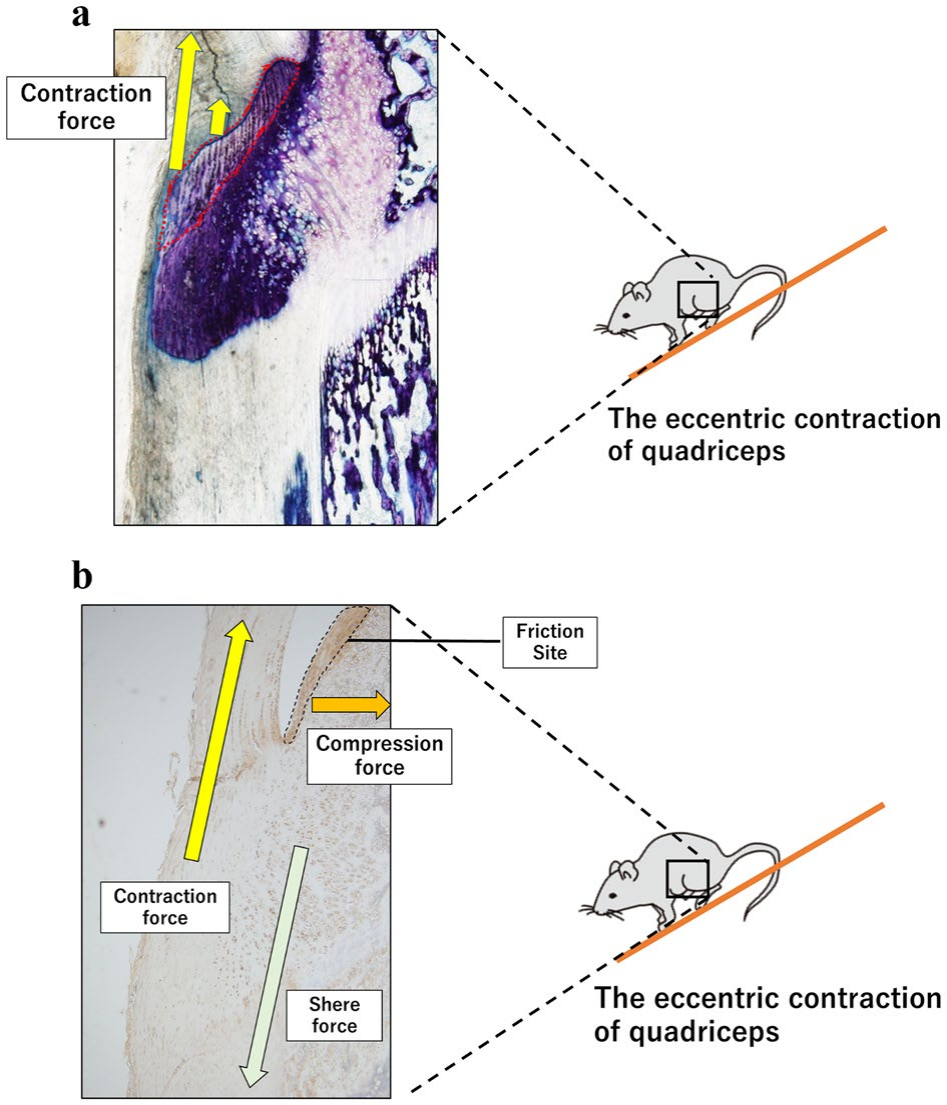
(**a**) The contraction on the tibial tuberosity during DR. From the viewpoint of the histological structures of the fibrocartilage portion (red circle), eccentric contractions during DR are speculated to pull on the anterior tibial tuberosity. (**b**) Three types of stress direction in the tibial tuberosity. The contraction force (yellow arrow) involves quadriceps contractions, and the deep region shows shear force (yellow-green arrow). Compression force (orange arrow) occurs in the posterior enthesis, the patellar tendon and articular cartilage are in close contact, and contraction can cause friction.

Thus, we speculated that the contraction force of the quadriceps on the tibial tuberosity induced a structural change in the fibrocartilage portion, depending on the strength of the contractions (Fig. 9a). Furthermore, the thickness of the periosteum in the DR group was significantly higher than that in the CO group. As the patella tendon penetrates the tibial tuberosity and connects to the periosteum, the thickness of the periosteum and the area of the superficial layer in the DR group were significantly higher than those in the CO group, respectively. Thus, it can be inferred that as the superficial region increases, not only is the periosteum thicker but the entire rough surface of the tibia is also larger.

The initial growth period was characterized by immature chondrocytes in the entire tibial tuberosity, and the condition of non-calcification was maintained around the matrix. The tibial tuberosity in the growing period was observed as superficial and deep regions distinct from the calcification condition, and in the late growing period, it was ossified throughout the tibial tuberosity. Therefore, the structural changes in the tibial tuberosity in terms of growth can be understood as the normal process of ossification of non-calcified cartilage. In contrast, PGE2 is considered to be an inflammatory cytokine^29^ that has been reported to inhibit chondrocyte differentiation^30^. Moreover, chondrocyte differentiation is controlled by the PI3K/Akt signaling pathway^31^. In general, the differentiation process of chondrocytes begins with mesenchymal stem cells changing into immature chondrocytes (small chondrocytes)^32^, and the cartilage matrix around the immature chondrocytes is calcified after they mature and become hypertrophic^33,34^. The PKA/PKC signaling pathway, however, inhibits chondrocyte differentiation, which is regulated by PGE synthesis^21^. In short, PGE2 can inhibit chondrocyte differentiation and calcification of cartilage tissues. In this study, the results of safranin O staining confirmed the deep region in each group, and several small chondrocytes were observed in DR, in contrast to that in CO. Thus, it is speculated that the tibial tuberosity in DR inhibits calcification and chondrocyte differentiation. This is supported by the wide variation in gene expression and morphometry in deep regions. The deep region was significantly higher in DR than in CO. Therefore, we can hypothesize that the tibial tuberosity in DR remains as cartilage tissue, which is speculated to be in a non-calcified condition. Furthermore, the genes for Cox2 and Ptges, which participate in PGE2 synthesis^35,36^, were significantly expressed in DR. As previously mentioned, PGE2 inhibits the calcification of cartilage tissues; thus, chondrocyte differentiation in the deep region is suppressed by eccentric contractions in the quadriceps, and the tibial tuberosity is speculated to grow as a result. This process is believed to involve PGE2 synthesis.

Significant levels of IL-6 were expressed in the tibial tuberosity in DR. IL-6 is widely known as an inflammatory cytokine and is involved in enthesis inflammation^9,37^. Therefore, it is considered that this DR protocol induces inflammatory cytokines in the tibial tuberosity. In addition, the tibial tuberosity in DR exhibited strong immunolocalization of substance P, widely recognized as a pain substance^38^, which is transmitted from free nerve endings. The main function of PGE2 is not only the suppression of chondrocyte differentiation but also downregulation of the pain threshold^38–40^. Thus, it appears that the tibial tuberosity in DR was caused by inflammation, pain, and prominence, as in OSD. Moreover, we believe that the mechanical function of the enthesis was involved in the strong reaction of substance P in the deep region and articular cartilage in DR. The fibrocartilage in the enthesis fluctuates, depending on the mechanical stress, and the fibrocartilage is known to protect the enthesis from tensile, shear, and compression forces^15,41^. We hypothesized that structural changes in the fibrocartilage are involved in the contraction force of the patellar tendon. The deep region was composed only of cartilage tissue, and the patellar tendon was not embedded. In this regard, it was speculated that this site experiences shear force instead of contraction force (Fig. 9b).

Gene expression revealed that eccentric contraction of the quadriceps resulted in upregulation of inflammatory cytokines and Ptges (involved in suppression of calcification). This is reflected by the deep region and articular cartilage showing strong immunoreactivity to substance P. Furthermore, the articular cartilage of the posterior patellar tendon in DR was shown to react with substance P; therefore, we believe that friction occurred in the anterior articular cartilage by quadriceps contraction as the patella tendon was compressed to the posterior by contraction. However, the environment for reducing mechanical stress promotes cartilage calcification^42,43^. It is believed that the symptoms of OSD are involved in suppressing calcification of the deep region and promoting calcification of the fibrocartilage portion.

The treatment or prevention of OSD is not yet established; however, it is quite clear that OSD is induced in the tibial tuberosity through repetitive eccentric contractions. A previous study reported the occurrence of eccentric contractions during DR in rats, which was implemented in the current study as well. The current research contributes to the elucidation of OSD structures, with the aim of paving the way for the treatment and prevention of OSD.

Nevertheless, the study has some limitations. OSD is particularly common in active adolescent boys; however, we only used rats in this study. Although the structure of the tibial tuberosity DR is relatively similar in humans and rats, it is unclear if they share the same recovery process. Further research is required to address this question.

## Data Availability

The data that support the findings of this study are available in this manuscript.
