# Author Correction: Eccentric contractions during downhill running induce Osgood‒Schlatter disease in the tibial tuberosity in rats: a focus on histological structures

**DOI:** 10.1038/s41598-023-38123-8

**Published:** 2023-07-10

**Authors:** Hirai Suito, Kaoru Fujikawa, Masafumi Ohsako

**Affiliations:** 1 Graduate School of Human Life Design, Toyo University, 1-7-11 Akabanedai, Kita-Ku, 115-8650 Tokyo, Japan; 2 Japan Society for the Promotion of Science Research Fellowships DC, Tokyo, Japan; 3 Department of Oral Anatomy and Developmental Biology, Showa University School of Density, Tokyo, Japan; 4 Graduate School of Health and Sports Science, Toyo University, Tokyo, Japan

Correction to: *Scientific Reports* https://doi.org/10.1038/s41598-023-36914-7, published online 18 June 2023

The original version of this Article contained an error in Affiliations 1 and 4.

Affiliation 1

“Graduate School of Human Life Design, Tokyo University, 1-7-11 Akabanedai, Kita-Ku 115-8650, Tokyo, Japan.”

now reads:

“Graduate School of Human Life Design, Toyo University, 1-7-11 Akabanedai, Kita-Ku 115-8650, Tokyo, Japan.”

And Affiliation 4

“Graduate School of Health and Sports Science, Tokyo University, Tokyo, Japan.”

now reads:

“Graduate School of Health and Sports Science, Toyo University, Tokyo, Japan.”

The original Article has been corrected.

